# The changes in clot microstructure in patients with ischaemic stroke and the effects of therapeutic intervention: a prospective observational study

**DOI:** 10.1186/s12883-015-0289-1

**Published:** 2015-03-15

**Authors:** Sophia N Stanford, Ahmed Sabra, Lindsay D’Silva, Matthew Lawrence, Roger HK Morris, Sharon Storton, Martyn Rowan Brown, Vanessa Evans, Karl Hawkins, Phylip Rhodri Williams, Simon J Davidson, Mushtaq Wani, John F Potter, Phillip A Evans

**Affiliations:** School of Medicine, Swansea University, Swansea, UK; NISCHR Haemostasis Biomedical Research Unit, Morriston Hospital, ABMU Health Board, Swansea, SA6 6NL UK; The Emergency Department, Morriston Hospital, ABMU Health Board, Swansea, UK; School of Applied Sciences, Cardiff Metropolitan University, Cardiff, UK; College of Engineering, Swansea University, Swansea, UK; Department of Haematology, Royal Brompton Hospital, Royal Brompton and Harefield NHS Foundation Trust, London, UK; Department of Stroke Medicine, Morriston Hospital, Swansea, UK; Norwich Medical School, University of East Anglia, Norwich, UK

**Keywords:** Ischaemic stroke, Clot microstructure, Fractal dimension, Biomarker, Thrombolysis, Aspirin

## Abstract

**Background:**

Stroke is the second largest cause of death worldwide. Hypercoagulability is a key feature in ischaemic stroke due to the development of an abnormally dense clot structure but techniques assessing the mechanics and quality of clot microstructure have limited clinical use. We have previously validated a new haemorheological technique using three parameters to reflect clot microstructure (Fractal Dimension (*d*_*f*_)) ex-vivo, real-time clot formation time (*T*_*GP*_) and blood clot strength (elasticity at the gel point (G’_GP_)). We aimed to evaluate these novel clotting biomarkers in ischaemic stroke and changes of clot structure following therapeutic intervention.

**Methods:**

In a prospective cohort study clot microstructure was compared in ischaemic stroke patients and a control group of healthy volunteers. Further assessment took place at 2–4 hours and at 24 hours after therapeutic intervention in the stroke group to assess the effects of thrombolysis and anti-platelet therapy.

**Results:**

75 patients (mean age 72.8 years [SD 13.1]; 47 male, 28 female) with ischaemic stroke were recruited. Of the 75 patients, 32 were thrombolysed with t-PA and 43 were loaded with 300 mg aspirin. The following parameters were significantly different between patients with stroke and the 74 healthy subjects: *d*_*f*_ (1.760 ± .053 versus 1.735 ± 0.048, p = 0.003), T_GP_ (208 ± 67 versus 231 ± 75, p = 0.05), G’_GP_ (0.056 ± 0.017 versus 0.045 ± 0.014, p < 0.0001) and fibrinogen (3.7 ± 0.8 versus 3.2 ± 0.5, p < 0.00001). There was a significant decrease in *d*_*f*_ (p = 0.02), G’_GP_ (p = 0.01) and fibrinogen (p = 0.01) following the administration of aspirin and for *d*_*f*_ (p = 0.003) and fibrinogen (p < 0.001) following thrombolysis as compared to baseline values.

**Conclusion:**

Patients with ischaemic stroke have denser and stronger clot structure as detected by *d*_*f*_ and G’_GP_. The effect of thrombolysis on clot microstructure (*d*_*f*_) was more prominent than antiplatelet therapy. Further work is needed to assess the clinical and therapeutic implications of these novel biomarkers.

## Background

Stroke is the most common life threatening neurologic disorder and is the second leading cause of death worldwide [1]. Abnormal coagulation in ischaemic stroke (IS) has been linked to altered clot microstructure [2]. This has been also reported in haemorrhagic stroke [3] but it is difficult to establish if this is due to the acute cerebrovascular event, an underlying hypercoagulability state or both. The structure of the fibrin clot is influenced by environmental and genetic factors [4]. These abnormal blood clots are composed of compact thin fibrin fibres and more resistant to lysis, which predispose to arterial thrombotic events. It has been shown that patients with ischaemic stroke have reduced clot permeability and impaired fibrinolysis similar to survivors of myocardial infarction [2,3,5]. Several coagulation factors including fibrinogen have been reported to be raised in stroke [6]. Nonetheless these factors and standard coagulation tests have limited clinical use in stroke [7,8] as they are functional time-based end point assays based on plasma incubation with exogenous reagents to activate the coagulation cascade [9,10]. Imaging techniques such as scanning electron microscopy (SEM) have also been used to investigate blood clots in IS [2] but have their own limitations. Coagulation kinetics and various properties of clots (mechanical strength, syneresis, opacity, and clot retraction) are associated with the structural characteristics of fibrin networks [11,12]. Of these parameters the viscoelastic properties of fibrin gels are the most sensitive measures of fibrin polymerization and clot structure, with numerous examples of their physiological importance [12]. Recent studies [13-16] have highlighted new biomarkers of haemostasis that measure these viscoelastic and structural changes. These biomarkers rely on the rheometrical measurement of blood as it clots and strict criteria for assessing when an incipient clot has formed. Such a criterion is provided by the Chambon-Winter Gel Equation [17,18], which accurately describes the Gel Point (GP) of samples of whole human blood [15,16]. From determination of this GP three parameters are measured: Time to the Gel Point or clot formation time (*T*_*GP*_), a quantitative measure of the fibrin microstructure of incipient blood clot (fractal dimension (*d*_*f*_)), and blood clot strength or elasticity at the gel point (*G’*_*GP*_). This study therefore aims to compare these biomarkers in patients with ischaemic stroke and healthy individuals and assess changes in clot microstructure ex-vivo following therapeutic intervention.

## Methods

### Study design

This is a prospective cohort study that compared blood clot structure ex-vivo in patients with first ever ischaemic stroke patients and healthy volunteers (control group).

### Patient population

Patients with a first ever acute IS were recruited upon presentation to the Emergency Department of a large teaching hospital (ABMU Health Board, Swansea, UK). Once a provisional diagnosis of stroke was made, strict inclusion criteria were applied: Adults (≥18 years) with IS were diagnosed by the treating clinician based on clinical history, examination and neuroradiology and checked by a member of the research team against WHO diagnostic criteria [19]. Subjects gave written fully informed consent, for those unable to consent due to lack of mental capacity assent was sought from their family members/friends (consultees) or a clinician independent of the study. Those who had had a previous stroke, were on anticoagulant therapy, or with disease states that are likely to affect coagulation (e.g. liver disease, malignancy, renal failure), or imminent death were excluded. Those who presented within 4.5 hours from onset of symptoms and met thrombolysis criteria received recombinant t-PA (Alteplase^©^), otherwise they were given 300 mg of aspirin after first blood sample irrespective of antiplatelet therapy at baseline. All patients received 300 mg at 24 hours unless CT showed intracerebral bleeding. We also recruited an age-matched control group of healthy subjects from the local population via various advertising means including posters, internal email or direct invitations to staff and patients’ relatives.

### Blood sampling and data collection

Once written informed consent was obtained clinical and demographic data were collected including ischaemic time (IT) defined as the start of symptoms to the time first blood sample was taken on arrival to the emergency department. A baseline venous blood sample was collected for both IS and healthy groups. A further 2 samples of blood were taken in the same manner in patients with stroke at 2–4 hours and at 24 hours after treatment to assess the effects of therapeutic intervention on clot structure. The first two mls of blood were discarded and the following tests were performed:

#### Rheometry (d_f_, T_GP,_ G’_GP_)

The haemorheological method used herein, which is performed using physiological unadulterated whole blood in a near-patient setting, has been described previously [14]. A 6.6 ml aliquot of whole blood was immediately loaded into a double-gap concentric cylinder of a TA Instruments AR-G2 (TA Instruments, New Castle, DE, USA) controlled-stress rheometer at 37°C (±0.1°C). The blood was immediately tested to obtain the GP, which marks the fluid to solid transition. The GP corresponds to the formation of the incipient clot in coagulating blood, whose sample-spanning fibrin network provides the mechanical basis for attaining a haemostatic function [15,20].

#### Laboratory tests

Further blood samples were taken for Full Blood Count (FBC), Prothrombin Time (PT), activated partial thromboplastin time (APTT) and fibrinogen levels. FBC samples were analysed on a Sysmex XE 2100 (TOA Medical Electronics) automated haematology analyser and routine clotting testing was undertaken using Sysmex CA1500 analyser. Fibrinogen concentrations were measured by ‘clauss’ method. The analysers were calibrated according to manufacturer’s instructions and fibrinogen calibration was checked against the 2^nd^ International Fibrinogen Standard Version 4 (NIBSC code 96/612). All reagents were obtained from Dade Behring.

### Statistical analysis

Descriptive analyses were performed to establish baseline characteristics of both groups. The normality of data distribution was assessed by normal probability plots and Shapiro–Wilk test of normality. Categorical variables are summarized using percentages and compared using chi-square tests while continuous variables are presented using mean and standard deviation (SD) unless otherwise stated. Differences were assumed to be significant at 5% level. A two-sample Student’s *t*-test was used to compare patients with stroke versus healthy subjects and stroke patients who were on antiplatelet drugs at baseline versus those who were not. Differences between the 2 groups were then evaluated by using analysis of covariance (ANCOVA) whilst controlling for the effect of smoking. The new biomarkers were also assessed in terms of ischaemic time comparing those who presented before and after 4.5 hours, which is the local cut-off for considering thrombolysis. Rheometric parameters (and standard tests) at baseline, 2–4 hours and 24 hours after therapeutic intervention were examined by one-way repeated measures analysis of variance (ANOVA). IBM SPSS statistical software package version 19.1 was used to perform the analysis.

### Determination of sample size

Sample size calculation was based on the difference for estimates of *d*_*f*_ in healthy and ischaemic stroke patients. Our preliminary data suggest that in subjects with confirmed ischaemic stroke we would expect a mean *d*_*f*_ of 1.78 with a standard deviation of 0.05. Using these values of an expected mean difference of 0.04 in *d*_*f*_, a power value of 0.9 and α = 0.05 suggested that 35 patients would be required. Consequently, we aimed to recruit at least 60 subjects to allow us to investigate secondary outcomes and explore any significant relationships between *d*_*f*_ and other markers of haemostasis and inflammation.

### Ethical approval

The study has been approved by the local ethics committee (South West Wales Research Ethics Committee).

## Results

123 patients with suspected first-ever ischaemic stroke were recruited between May 2012 and February 2014. 48 patients were excluded for various reasons (18 had stroke mimics such as Bell’s palsy, sepsis and brain tumour; 8 transient ischemic attacks (TIA); 8 had a haemorrhagic stroke; 4 previous strokes; 6 found to have been given loading aspirin prior to blood collection; 1 renal failure and 3 cancers). Seventy-five patients (47 men and 28 women; mean age, 72.8 ± 13.1 years) were confirmed to have ischaemic stroke and included in this analysis. Their Baseline characteristics are presented in Table 1. Stroke patients were matched for age and sex with 74 healthy subjects (42 men and 32 women; mean age, 70.6 ± 7.1 years) recruited from local populations but there were more current smokers in the stroke group than healthy controls (24% versus 8.1%, p = 0.01).

**Table 1:** **Baseline characteristics of patients with stroke (n = 75)**

**Age, mean ± SD**	**72.8 ± 13.1**
**Sex: Male/female**	47/28
**Current smoker**	18 (24%)
**Hypertension**	49 (65.3%)
**Ischaemic heart disease**	21 (28%)
**Atrial fibrillation**	18 (24%)
**Diabetes mellitus**	17 (22.7%)
**Previous TIA**	11 (14.7%)
**Hyperlipidaemia**	30 (40%)
**Antiplatelet use**	34 (45.3%)
**Aspirin**	27
**Clopidogrel**	3
**Both drugs**	4
**Statin use**	27 (36%)
**Glucose (mmol/L)**	6.6 ± 1.9
**Creatinine (μmol/L)**	95.7 ± 27.1
**D-dimer (ng/mL)**	199 [107–463]
**C-reactive protein (mg/L)**	3 [1-9]
**TOAST Classification:**	
Large artery	22
Cardio-embolic	14
Small vessel (lacunar)	31
Other determined aetiology	1
Undetermined aetiology	7

Values are presented as percentages, mean ± SD or median (interquartile range).

The means (SD) of rheometric measurements and standard tests for healthy subjects and IS patients at baseline are presented in Table 2. *d*_*f*_, *G’*_*GP*_ and fibrinogen were significantly lower in healthy subjects (p = 0.003, p < 0.00001, p < 0.00001 respectively) whereas APTT was shorter in patients with IS (p < 0.0001) as shown in Table 2, which continued to be significant after controlling for smoking status using ANCOVA.

**Table 2:** **Rheometric and haematological tests for stroke patients (baseline) and healthy subjects**

	**Stroke**	**Healthy**	**P**
***d***_***f***_	1.760 ± .053	1.735 ± 0.048	0.003
***G’***_***GP***_ **(Pa)**	0.056 ± 0.017	0.045 ± 0.014	0.00009
***T***_***GP***_ **(secs)**	208 ± 67	231 ± 75	0.05
**Hb (g/dl)**	14.2 ± 1.7	14.3 ± 1.3	0.63
**Plt (×10** ^**9**^ **/l)**	253 ± 78	246 ± 50	0.52
**HCT (g/l)**	0.42 ± 0.04	0.42 ± 0.04	0.44
**PT (secs)**	10.6 ± 0.6	10.6 ± 0.6	0.50
**APTT (secs)**	24.3 ± 2.2	25.9 ± 2.1	0.00003
**FBG (g/l)**	3.7 ± 0.8	3.2 ± 0.5	0.000003

Values are presented as mean ± SD.

Abbreviations: *d*_*f*_ fractal dimension; *G’*_*GP*_ elasticity at the gel point; *T*_*GP*_ real-time clot formation time; Hb hemoglobin; Plt platelet count; HCT haematocrit; PT prothrombin time; APTT activated partial thromboplastin time; FBG Fibrinogen.

*p < 0.05 assumed significant as detected by *t*-test analysis.

At baseline 34 stroke patients were on antiplatelet therapy (27 on 75 to 300 mg of aspirin only, 3 on 75 mg of clopidogrel only and 4 on both agents). *d*_*f*_ and PT were significantly different when we compared stroke patients who were on regular antiplatelet therapy at baseline versus those who were not (*d*_*f*_ = 1.74 ± 0.050 versus 1.77 ± 0.053, p = 0.01; PT = 10.8 ± 0.7 versus 10.5 ± 0.5, p = 0.01 respectively). Patients with IT <4.5 hours had lower *d*_*f*_ and *G’*_*GP*_ values as compared to those who presented later (*d*_*f*_ = 1.75 ± 0.05 versus 1.77 ± 0.05, p = 0.10; G’*GP* = 0.053 ± 0.017 versus 0.060 ± 0.017, p = 0.11).

**Figure 1:**
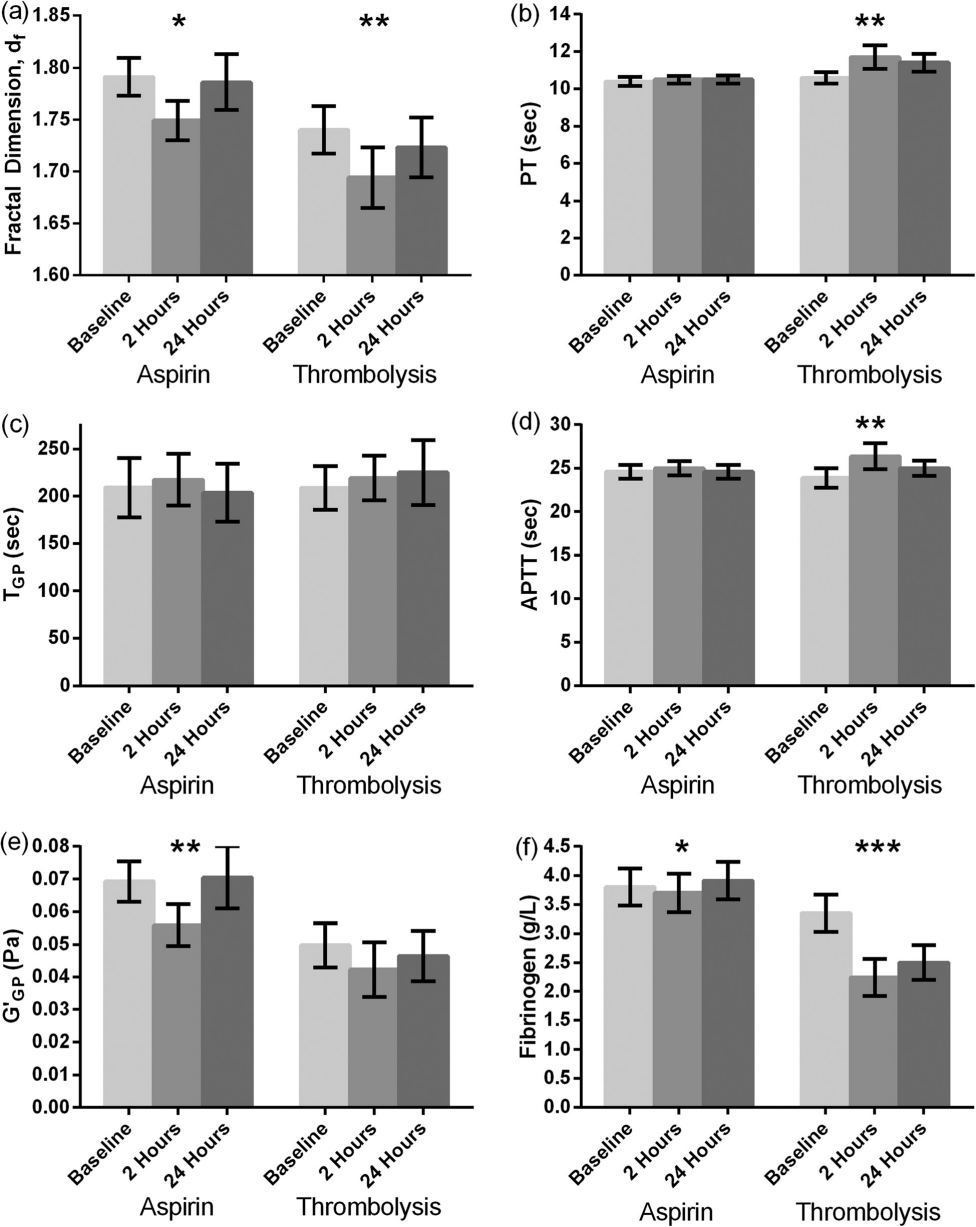
**Bar chart showing rheometric and clotting tests for both groups of patients (receiving aspirin or thrombolysis).** Bars plotted represent mean and 95% confidence interval of *df* **(a)**, *TGP* **(b)**, *G’GP* **(c)**, PT **(d)**, APTT **(e)** and fibrinogen **(f)** for both groups at baseline, 2–4 hours and 24 hours after treatment. Within group differences were compared using ANOVA. *p < 0.05, **p < 0.01, ***p < 0.001.

Of the 75 patients, 32 were thrombolysed and 43 were loaded with 300 mg aspirin. Blood results for the 3 sampling points were available for 41 patients (21 in the thrombolysis group and 20 in the aspirin group). Ischaemic time was significantly shorter in the thrombolysis group as compared to those who received aspirin only ((mean 2:34 ± 1:03 vs 7:12 ± 6:15, p = 0.001). As can be seen in Figure 1, ANOVA revealed that following the administration of aspirin *d*_*f*_ (p = 0.02), *G’*_*GP*_ (p = 0.013) and fibrinogen (p = 0.014) were significantly lower, whilst with thrombolysis *d*_*f*_ (p = 0.003), fibrinogen (p < 0.001), PT (p < 0.01) and APTT (p < 0.01) were significantly different. This analysis was repeated for patients who were aspirin-naive at baseline and showed similar trends.

## Discussion

**Figure 2:**
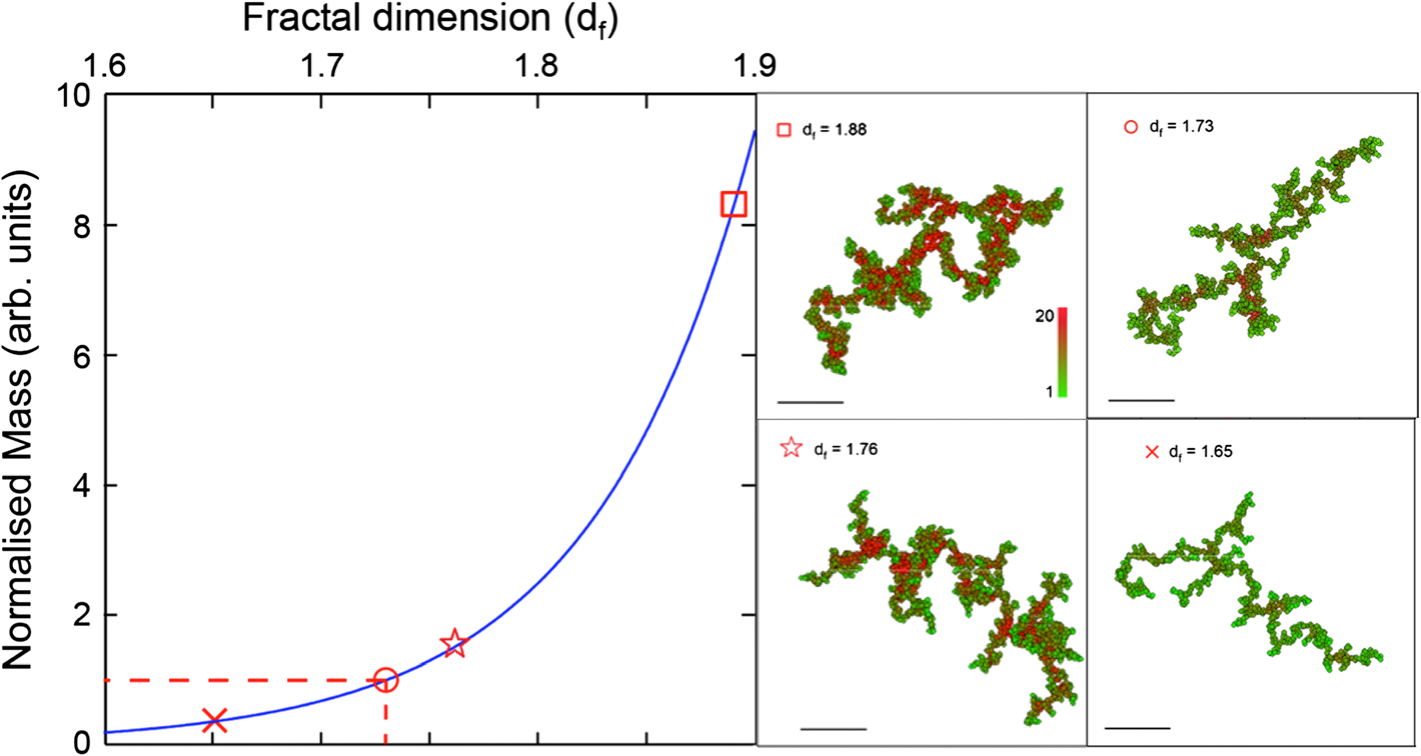
**The non-linear relationship between the fractal properties of the incipient fibrin clot measured by** ***d***_***f***_ **and the amount of mass, incorporated into the structure.** The mass value on the y-axis is normalised for the healthy value of *d*_*f*_ (=1.73). Illustrations of different incipient clot microstructures at particular values of *d*_*f*_ are provided (cross = 1.65, circle = 1.73, star = 1.76 and square = 1.88 respectively).

**Figure 3:**
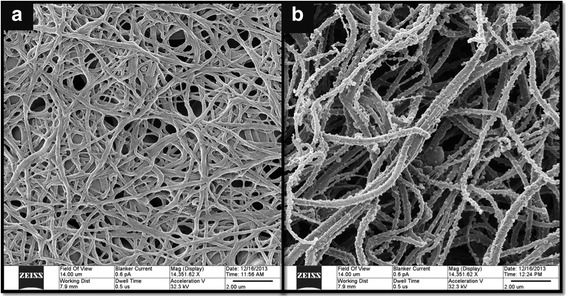
**Representative SEM micrographs of fully formed blood clots taken from the same individual before (a) and 2 hours after thrombolysis (b).** The images show a significant change in the clot microstucture characteristics, where at point A the blood clot was denser with more branching points corresponding to a *d*_*f*_ value of 1.71. At point B, although the clot seems to have thicker fibre width it was more porous and corresponded to a lower *d*_*f*_ value of 1.66. The scale bar is similar and applies to both images. The patient was on aspirin at baseline.

Abnormal coagulation plays a pivotal role in the pathophysiology of ischaemic stroke, as demonstrated by the dense clot structure in the stroke group [2,21]. Current coagulation tests are kinetic markers of coagulation but do not quantify the abnormal clot structure seen in the disease. They are at best described as adjuncts to clinical care. However, there is limited information on the biophysical properties of fibrin clot structure and strength in stroke. In this study we describe for the first time rheological markers that detect an abnormal clot structure. This Gel Point-derived new biomarker is composed of three parameters: clot formation time, clot microstructure and clot strength as measured by *T*_*GP*_, *d*_*f*_ and *G’*_*GP*_. Rheological analysis revealed that clot structure and strength as measured by *d*_*f*_ and *G’*_*GP*_ were significantly different between the stroke and healthy populations. Although the changes in *d*_*f*_ may seem small, it is very important to recognize that *d*_*f*_ has a non-integer value with very narrow range (in this study 1.59 to 1.88) and non-linear relationship with the amount of fibrin mass incorporated within the incipient clot (Figure 2), which means substantial increases in mass are required to generate even small increments of *df* [20,22,23]. Hence a clot with a *d*_*f*_ value of 1.760 (patients with stroke) incorporates 40% more mass than a clot formed with a *d*_*f*_ of 1.735 in healthy subjects [15]. SEM images of a stroke patient were consistent with *d*_*f*_ changes before and after thrombolysis as seen in Figure 3. These images were prepared and scanned as previously reported [14]. Although the mean fibre width was higher after thrombolysis, the clot was more porous with lesser branching points. *d*_*f*_ measurement was done simultaneously and the change in *d*_*f*_ was consistent with the change in fibre width. *d*_*f*_ decreased from 1.71 at baseline to 1.66 after thrombolysis. Another interesting observation is the change in the fibrin fibre surface following thrombolysis. Several proteins such as plasminogen, t-PA, albumin, a2AP and PAI-1 bind to fibrin, which may explain the rough surface of these fibres following thrombolysis as seen in panel B (Figure 3). The larger knobs look more like microparticles, which are phospholipid microvesicles containing certain membrane proteins derived from their parent cells [24] but this needs further investigation.

Fibrinogen levels were higher in the stroke group when compared to the healthy, which has been reported in previous studies [25,26]. Although a significant difference in APTT was observed between the stroke and healthy populations, both values were within the normal reference range and not clinically significant.

We observed lower values of *d*_*f*_ in patients who were on antiplatelet therapy at baseline as opposed to patients who were aspirin-naive. This is an important finding suggesting this new biomarker can detect the effect of prophylactic antiplatelet drugs. Additionally the effects of therapeutic intervention were detected by a reduction in *d*_*f*_, *G’*_*GP*_ and fibrinogen levels after the administration of loading dosage of aspirin. This trend was observed in all patients whether on aspirin or not at baseline. Aspirin primarily acetylates platelet cyclo-oxygenase-1, which results in the irreversible inhibition of thromboxane A_2_ production and therefore reduction in platelet aggregation. However, at higher doses it also has other effects including acetylation of fibrinogen and prothrombin and other coagulation factors [27-29]. It therefore has a role in modulating clot structure and this was measurable by our global haemorheological markers of clot structure. It was surprising that the values of all tests investigated returned back to baseline values at 24 hours. Cerebral Infarct progression has been characterized by three phases namely the acute ischaemic phase (minutes to hours after stroke onset), subacute neuroinflammatory phase (hours to days) and chronic regenerative phase (days to months) [30]. Therefore a progressing inflammatory response during the subacute phase or even a stronger inflammatory response due to reperfusion-induced injury [31] may explain the reverting of *d*_*f*_ and G’_GP_ values at 24 hours following an initial response to therapy. Treatment with thrombolysis caused a reduction in fibrinogen levels and some prolongation of PT and APTT at 2 hours post treatment. Recombinant t-PA not only breaks down fibrin but also has an effect on fibrinogen, factors V and VIII [32]. This could explain the changes in standard markers of coagulation post thrombolysis but considering testing was done 2 hours later it was expected that these changes will not be significant because t-PA has a very short half life [33]. Our haemorheological markers not only confirmed the changes due to thrombolysis as revealed by standard tests but also detected the subtle changes following aspirin.

Although non-significant, our study showed that patients who presented early with an ischaemic time of less than 4.5 hours had lower *d*_*f*_ and *G’*_*GP*_ values as compared to those who presented later. One explanation for this could be that longer ischaemic time results in stronger and denser blood clot. Various studies in myocardial infarction have shown that shorter ischaemic time has better outcomes [34-36]. Silvain et al. 2011 found that in clots obtained from patients undergoing primary PCI for myocardial infarction the platelet and fibrin fibre components changed rapidly with respect to ischaemic time. With time the thrombus evolves from a ‘fresh’ platelet rich one to an ‘old’ fibrin rich thrombus [37,38]. This could also underpin the effectiveness of fibrinolytic treatment within the first few hours only. Furthermore, this may be the period when the clot has a small and accessible amount of fibrin fibres and when fibrinolysis has not yet been overcome by the intense fibrin fibre formation as seen in ‘old’ clots [38]. Additionally, the fast accumulation of fibrin fibres in the thrombus followed by its stabilisation by FXIII cross-linking is likely to increase clot stiffness leading to reduced fibrinolysis [5]. This may be similar in ischaemic stroke, which is reflected by the lower *d*_*f*_ measurements for patients who presented early and were thrombolysed.

One limitation of this study is related to the characteristics of patient population such as ethnicity and smoking. As all patients in this study were Caucasians, the results from this study may not be generalizable to other ethnic groups. Smoking affects coagulation [39] but in our study the difference between the 2 groups remained even after adjustment for smoking status. Additionally haemostasis may be affected by menopausal status [40] but we do not believe this had any effect on the findings reported because 83% of patients in our study were older than 65 years and only one premenopausal woman was recruited. The small number of patients is another limitation and a larger study is needed to confirm these findings and explore the role of these biomarkers in guiding therapeutic intervention. Thirdly, despite the strict inclusion and exclusion criteria stroke is a heterogeneous condition in terms of aetiology and severity, which is illustrated by the difference in rheological parameters between the thrombolysis and aspirin groups. This is an important observation and any future study should delineate between these groups (superacute and acute stroke). The stroke group was compared to healthy subjects and hence the differences observed may be due to an underlying hypercoagulable state due to other co-morbidities rather than just having an acute stroke. Despite these limitations, this new haemorheological biomarker has the potential to inform clinical management decisions as it provides more insight of the combined effect of clotting factors and cellular component in terms of fibrin clot structure and strength, which is a more comprehensive assessment of coagulation. Because of these unique features, incorporating this blood-based biomarker into risk prediction scores that are based on clinical factors only may overcome some of their limitations and improve their precision in identifying patients at high risk for stroke (e.g. CHA_2_DS_2_VASc score) [41] or predicting poor outcome in TIA (ABCD2 score) [42] and acute ischaemic stroke (e.g. the SNARL score) [43].

## Conclusions

Our findings using rheological analysis provide new information in terms of biophysical properties of the fibrin clot structure and strength in stroke. Furthermore, for the first time we can accurately quantify clot structure based on rheological measurements. This new information evaluates another aspect of the clotting system and may potentially aid in risk prediction and managing patients with stroke. In conclusion, acute ischaemic stroke patients have a hypercoagulable state resulting in a structurally denser and stronger clot as evaluated by *d*_*f*_ and *G’*_*GP*_ values. The changes following therapeutic intervention were more prominent following thrombolysis. Based on the results from this study we plan to undertake a larger study to assess if the new biomarkers are useful in identifying patients at high risk for stroke, or predicting successful recanalization and bleeding risk following thrombolysis and therefore evaluating the potential to guide therapeutic intervention.
